# A study on the influence of situations on personal avatar characteristics

**DOI:** 10.1186/s42492-024-00174-7

**Published:** 2024-09-23

**Authors:** Natalie Hube, Melissa Reinelt, Kresimir Vidackovic, Michael Sedlmair

**Affiliations:** 1 The Visualization Institute, University of Stuttgart, Stuttgart, 70569 Germany; 2 Mercedes-Benz AG, Sindelfingen, 71063 Germany; 3 Hochschule der Medien - University of Applied Sciences, Stuttgart, 70569 Germany

**Keywords:** Avatars, Virtual reality, User study, Social situations

## Abstract

Avatars play a key role in how persons interact within virtual environments, acting as the digital selves. There are many types of avatars, each serving the purpose of representing users or others in these immersive spaces. However, the optimal approach for these avatars remains unclear. Although consumer applications often use cartoon-like avatars, this trend is not as common in work settings. To gain a better understanding of the kinds of avatars people prefer, three studies were conducted involving both screen-based and virtual reality setups, looking into how social settings might affect the way people choose their avatars. Personalized avatars were created for 91 participants, including 71 employees in the automotive field and 20 participants not affiliated with the company. The research shows that work-type situations influence the chosen avatar. At the same time, a correlation between the type of display medium used to display the avatar or the person’s personality and their avatar choice was not found. Based on the findings, recommendations are made for future avatar representations in work environments and implications and research questions derived that can guide future research.

## Introduction

**Fig. 1:**
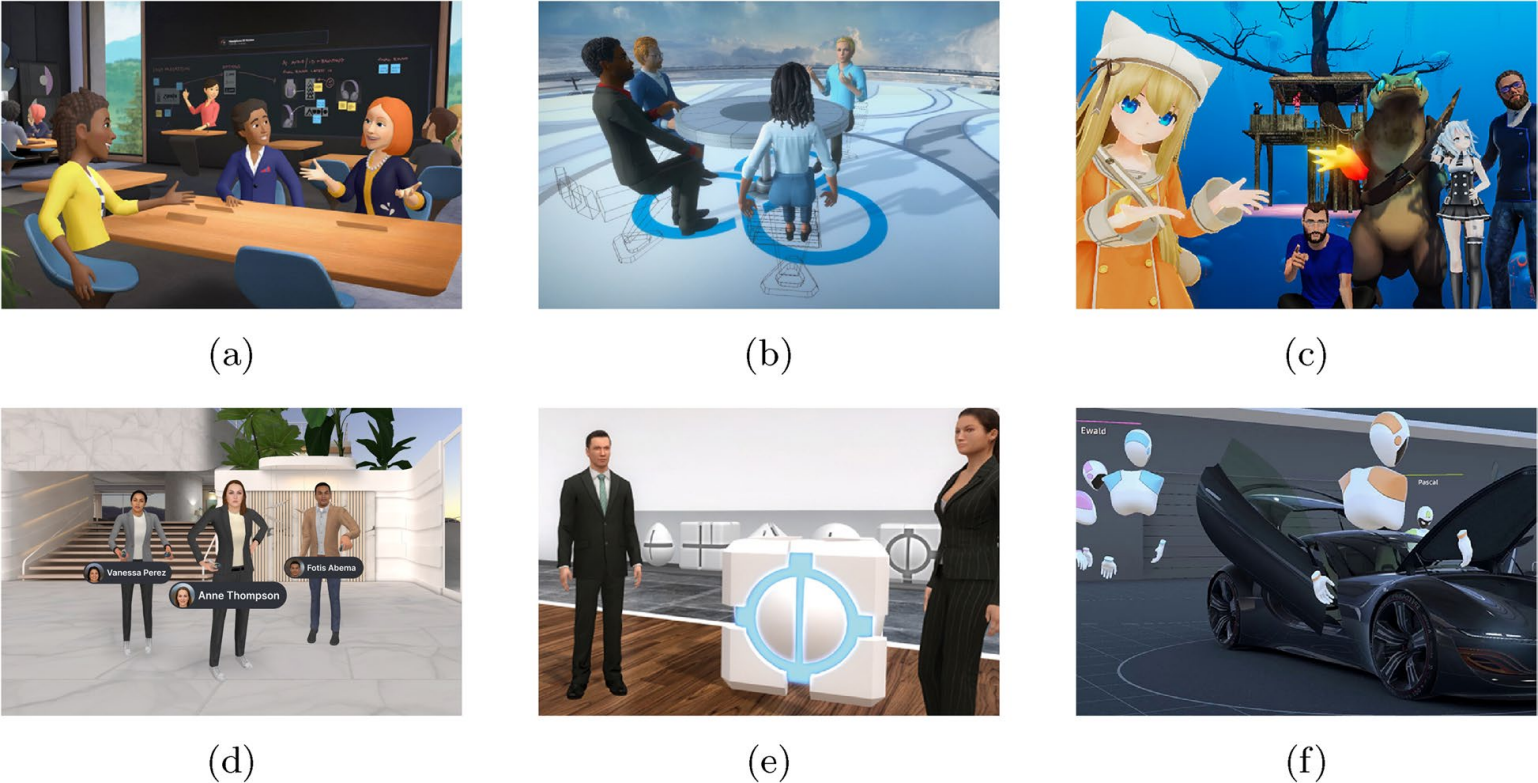
Consumer and work applications use a variety of different anthropomorphic avatars. **a** A stylized avatar with arms and missing lower part of the body [2]; **b** Stylized human avatars used in Vive Sync [3]; **c** Non-human and cartoonish avatars with a wide variety of different appearances [4]; **d** Arthur [8] with robot-like avatars with labels above their heads; **e** Lifelike human avatars used in Tricat Spaces [9]; **f** VRED [5] with robot-like avatars standing around a car

Avatars are widely used in collaborative virtual environments (CVEs) to represent users, allowing remote collaboration in virtual immersive spaces [1]. A broad spectrum of CVEs that support avatars [2–5] exists, each offering different levels of avatar realism and resemblance to humans (Fig. 1). Entertainment-based apps, such as VRChat [4] lean towards more stylized or even nonhuman avatars because users might prefer a less lifelike look in virtual settings [6]. However, applications designed for work contexts tend to feature avatars that appear more humanlike. Vive Sync [3], for example, offers stylized but professional-looking avatars, whereas other tools might opt for robotic or abstract designs [5]. Users engaging with these platforms may not always want a lifelike representation and could favor a stylized version that does not directly mimic their appearance [6]. However, in the work context, these expectations may shift [7].

In work settings, there is a clear tendency towards lifelike avatars [9]. Vive Sync [3] creates avatars from photos that, while colorful and stylized, are intended for work meetings. When lifelike human avatars are not an option because of the specific use cases in which they are used, abstract or robot-like avatars come into play, as seen in VRED [5], especially for virtual reality (VR) applications in work scenarios. As these avatars can make it challenging to recognize colleagues or foster social connections, finding suitable representations is crucial [7]. Despite the availability of different avatar systems, a standardized approach to representing avatars does not exist, which varies in realism and human likeness across different tools, lacking consistent guidelines for avatar usage in specific contexts.

This study aims to explore whether preferences for self-avatars, focusing on face geometry and texture style, change depending on the social setting. From the automotive industry standpoint, we are interested in how avatar choices differ between work and leisure situations. Harry [10] noted that workplace and leisure situations differ distinctly, a view supported by Dubin [11]. Further research highlights the divergent nature of the work and leisure domains [12]. Moreover, we examined how personal traits influence avatar choices and the effect of face geometry and texture material on avatar selection, as these elements affect an avatar’s appearance, ranging from life-like to stylized looks [13, 14].

To gain a deeper insight, three studies were conducted with 91 participants using a tool that allows avatar customization through face geometry and texture materials based on personal photos. The first study involved 71 employees from an automotive company, followed by a study of 20 people not associated with the company, to identify the differences between internal and external groups. A subsequent VR study with 17 participants provided further insights.

This research explores avatar preferences in professional vs leisure contexts. The findings suggest a trend towards favoring avatars that closely resemble human features (i.e., more verisimilitude) in work settings as opposed to leisure settings, where the preference shifts towards more stylized or abstract representations. These preferences were observed across different display media and backgrounds. This paper discusses the outcomes of the avatar selection studies with the aim of contributing to the ongoing discussion on CVE designs for future virtual environments (VEs).

The review of related work was narrowed to the aforementioned areas, ensuring a focused exploration of avatar selection influenced by the situation and representation styles. These factors are important for understanding and contextualizing the analysis of avatar selection within CVEs. It is crucial to understand how individuals perceive themselves and their avatars in a VE [7, 15, 16], because specific avatar forms influence social entities [17]. For avatars used in collaborative tools, the collaboration context must be considered when determining an appropriate representation style [18].

## Avatar representations in immersive environments

In immersive VEs, avatars are crucial because they act as digital proxies for users, enabling communication and mirroring users’ actions and psychological states [19]. These digital proxies allow for social experiences in VEs that mirror interactions in the physical world [20, 21].

The term avatar has been explored extensively in research, and is generally recognized as the digital persona of a real-world entity, sometimes even as a simple 2D icon in a digital meeting [22–25]. A key distinction in the literature is between *agents*, which are computer operated, and avatars, which are user controlled [26–29]. This study defines an avatar as a 3D, real-time, and manipulable digital representation of a user in a VE, specifically focusing on human-like avatars.

Studies have highlighted the role of avatars in enhancing social interaction within CVEs despite the variance in avatar fidelity [30–33]. The quality of an avatar, including its behavioral realism, sense of body ownership, and trustworthiness, influences social interactions [34–37]. Research has also delved into how the representation of collaboration partners and self-representation in CVEs affects collaboration, often without distinguishing between use cases or levels of stylization [38, 39].

The presence and realism of avatars are known to aid the sense of social presence in CVEs [35, 40], thereby enhancing communication and the overall immersive experience [41, 42]. The provision of personalized avatars has been suggested to further improve social interactions [38, 43]. However, the preference for lifelike vs stylized avatars in different scenarios remains an area for further exploration.

In addition, recent studies have emphasized the importance of avatars in fostering creativity within VEs. Liu et al. [44] systematically reviewed the creativity-boosting effects of user and contextual digital representations in VEs and highlighted the significant roles of embodiment and presence in creative processes.

## Avatar customization and psychological impact

Research on avatar customization and its psychological effects underscores the significant interplay between personal identity, technological capabilities, and situational contexts. Research emphasizes the users’ desire for nuanced avatar representations to accurately reflect their identities, which is influenced by social VR contexts [45, 46]. These findings highlight the need for flexible avatar customization options to accommodate diverse user preferences. Technological advancements have expanded the possibilities of avatar stylization, indicating that the availability of advanced customization tools influences the design choices of users [47]. This highlights the role of technology in facilitating personalized digital representations.

The psychological impact of avatar appearance, as explored by multiple researchers, shows that user experiences and preferences for avatar realism vs stylization are shaped by first impressions, therapeutic objectives, and gameplay contexts [48–51]. These studies demonstrate how psychological responses to avatars vary based on their use in specific situations, thereby affecting users’ choices in avatar design.

Overall, this study underscores the importance of considering both technological capabilities and situational factors when designing avatars that support identity expressions and positive user experiences in virtual spaces.

## Situational fidelity of avatars

To explore how different situations affect avatar representations in CVEs, it is essential to understand the elements that affect avatar appearance. The concept of *fidelity* is commonly used to describe how closely virtual experiences mimic real-life experiences, and is defined as the objective precision in replicating real-world experiences in the virtual domain [52]. Although some argue that a moderate level of visual accuracy is generally sufficient [53], the necessity for a high resemblance between an avatar and its owner may vary depending on the context [54].

Situational fidelity refers to the specific circumstances that influence actual behavior [55], and is grounded in semantic theory as a relational meaning [56]. However, a situation extends beyond mere symbolic representation [57], with social behavior emerging from the interplay between the situation and an individual’s interpretation by it integrating both objective and subjective elements [12, 58, 59]. Here, it is considered that a situation is any state that influences an individual at a given time and place, shaped by their behavioral patterns. Despite the importance of situational context, its impact on avatar fidelity has not been extensively studied.

The findings from recent research on user perceptions of different monitoring modalities during high-fidelity simulations may offer insights into how situational fidelity impacts the user experience with avatars. Akbas et al. [60] conducted a semi-quantitative analysis of participants’ perceptions of three monitoring modalities, including a visual patient avatar, highlighting how different representations might affect users’ situational awareness and engagement. Moreover, the development of Fydlyty, a low-fidelity serious game for cultural-competence education, suggests that the role of graphical fidelity in learning and user engagement can vary based on the educational context [61].

By incorporating these insights, the subtle impacts of situational fidelity on avatar representations can be better understood, suggesting directions for future research on avatar design and implementation in CVEs.

## Virtual character design

Studies have identified features as key to how virtual characters are perceived, emphasizing the role of face geometry and textures in their portrayals [13, 14]. This is related to the uncanny valley phenomenon, in which a reduction in realism may actually make a character more appealing and less eerie [17, 62]. Stylization, or varying degrees of deviation from lifelike representations, has been noted to affect how positively characters are viewed, with certain combinations potentially leading to adverse reactions [13].

This study aims to examine how different levels of stylization in shape and material, which are crucial for a character’s visual identity, impact viewer perception in various situational contexts. This was explored by contrasting a lifelike avatar with a highly stylized one and observing how they are received in different situations. Following the insights of Zell et al. [13], the shape and material were observed as the primary influencers of a virtual character’s appearance and these principles were applied to craft both lifelike and stylized avatar models. Considering recent findings on the effects of virtual characters’ body types on perceived believability [63, 64], this investigation also considers the broader implications of character design on user interaction and engagement.

In this study, two distinct virtual avatar styles of the user, termed *lifelike* and *stylized*, are compared across different situational contexts The aim is to create personalized avatars that are well-suited for their specific uses, whether for work or leisure. Recent research that highlights face geometry and texture style as two pivotal factors shaping a virtual character’s appearance were used [13, 14]. These elements determine whether an avatar appears *lifelike* or *stylized*.

In detail, both terms are referred to as

- *Lifelike* Avatars that emulate human-like human features including proportional facial geometry and naturally textured skin.
- *Stylized* These avatars break away from traditional human features, offering exaggerated forms and creative textures that might include abstract designs or vibrant, non-natural colors.

**Table 1:** Overview of the three studies, showing the devices used to display the situational conditions and individual avatar representation, as well as the number of participants from within and outside an automotive company

Study	I	II	III
Situation	Condition *leisure* vs *work*		
Device	Desktop		VR HMD
Setting	Within company	Outside company	Within company (subset)
Participants	71	20	17
Findings	Difference between condition *work* and *leisure* regarding face geometry and texture fidelity	Similar to Study I, no evidence for a difference regarding the field of work	No evidence for a difference between immersive and non-immersive environment

In total, we conducted the study with 91 participants

*HMD* Head mounted display

## Methods

To explore how different situations influence what kinds of avatars people prefer, a set of subsequent studies were combined. These studies required participants to adjust their individual avatars by focusing on two main aspects, *face geometry* and *texture material*, in two distinct settings, *work* and *leisure*. Three separate studies were conducted: the first with participants working at an automotive company (Study I), the second with participants not associated with the company (Study II), and the third follow-up study within a fully immersive VR setting (Study III). A summary of these studies can be found in Table 1. In the following sections, the details on how the individual avatars are created, the tool used to adjust the avatars, the design of the studies, and the overall setup are provided.

**Fig. 2:**
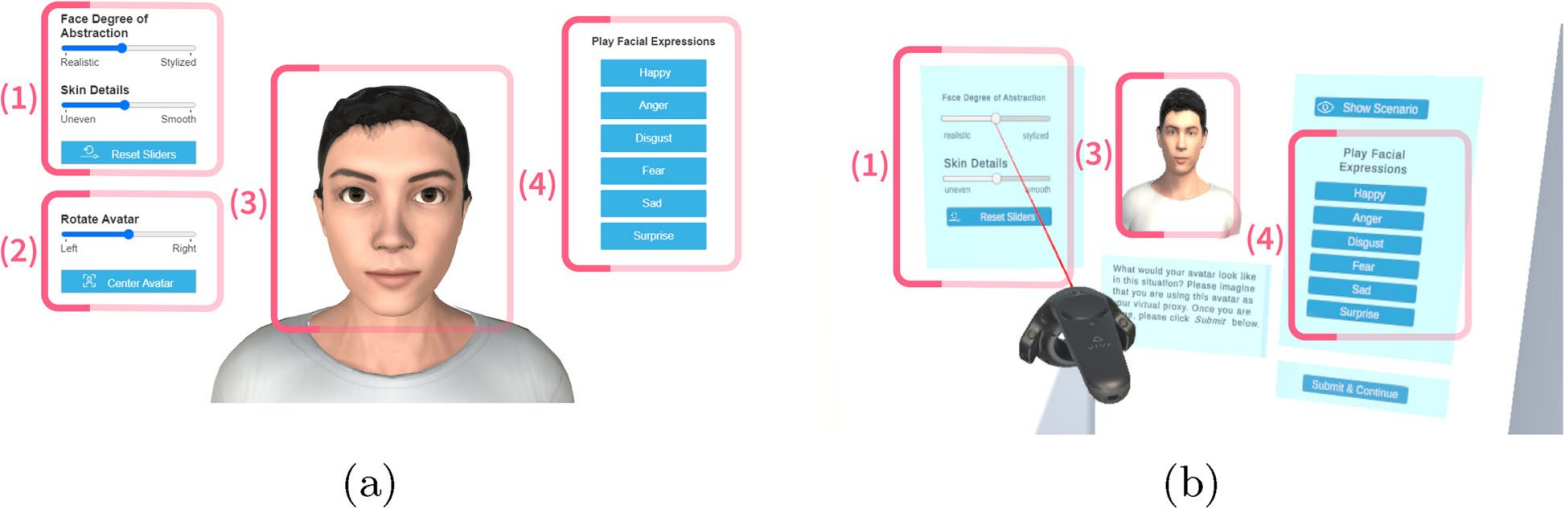
Desktop View (**a**) and VR View (**b**) showing the individual representation. The interface consists of four components: (1) slider interaction to adjust face geometry and texture material, (2) *[only desktop]* elements to rotate and center the avatar, (3) avatar representation, (4) buttons to test facial expressions on the avatar

### Prototype development for avatar customization tool

To study how different scenarios affect avatar choice, a prototype tool that allows users to adjust their individual avatars was developed. Additionally, an individual avatar for each participant was created from a photograph provided in advance. The tool allows users to continuously morph between a lifelike and stylized avatar. This tool offers a slider feature, making it easy for users to shift between looks that are more lifelike or stylized in nature. The slider tool is used as a method for easily adjusting the avatar looks, allowing immediate visual feedback (Fig. 2), as this is a well-known method for adjusting the appearance of avatars [65].

#### Preparation of personalized avatars

This study required the design of different levels of stylization of the same character based on the participants’ 2D photos. Stylization refers to the abstraction of representations to a certain degree. The focus is on the parameters of face geometry and texture material [13], which are compared in this study because these are the most important variables for determining avatar stylization. Face geometry refers to the shape of a face and whether it has more lifelike or stylized features. Texture refers to the skin texture. The aim when selecting the two parameters is to see which factors particularly influence the appearance and perception of the individual avatars.

**Fig. 3:**
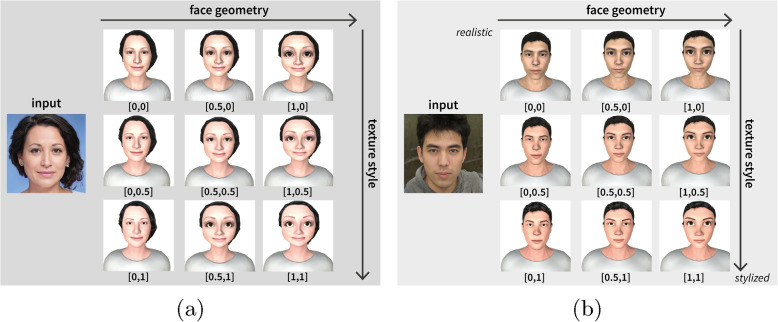
Sample visualization for a female (**a**) and male (**b**) avatar sorted by face geometry and texture style, from left to right and top to bottom (lifelike $\rightarrow$ stylized). Specific values are mentioned in the brackets below as *[face geometry, texture material]*. The picture input used in this example are styleGAN images from This Person Does Not Exist created by Karras et al. [67]

As the user study was prepared, individual avatars that required a few manual adjustments were created. Another requirement for the output model is that it must be renderable in a web browser. *Character Creator 3* [66] was used to create and customize the virtual characters. The software enables easy creation of a lifelike base model for the face geometry and texture material, from which 3D artists modeled and adjusted the stylized avatar to fit the lifelike model (Fig. 3). Because the focus is on the face geometry, the avatar was left with the rudimentary hair model depicted in the photo. A skilled artist created a stylized texture based on an input image.

To continuously morph between the lifelike and stylized models, blended shapes were created for face geometry and a texture blend shader for skin details. Additionally, for each avatar, four universal facial emotions-anger, happiness, sadness, and surprise were applied [68]-in addition to the neutral expression. Furthermore, random blinking and eye movements were added to increase the feeling of social presence of the avatar [69, 70].

#### System description

The online study was developed using the *Unity* [71] WebGL export for avatar interaction, which was then embedded on a website. The website was accessible through a VPN connection, and data storage was managed using an MSSQL database.

### Study design & setup

A between-subjects design was used to compare the values chosen for the avatar parameters, face geometry, and texture material between the two conditions: (A) *leisure* and (B) *work*. The situational condition is the independent variable.

#### Conditions

A sequence of three scenarios was designed for each condition (Table 2). The condition *leisure* illustrates situations involving public and private social interactions with familiar people as well as strangers. By contrast, in condition *work*, a situation targeting social interaction with known and unknown peers of different hierarchies in a working environment is presented. During the study, the participants were asked to immerse themselves in these situations while adjusting their personal avatars. The participants could look at the assigned set of situations at any time.

**Table 2:** Shortened situation descriptions used for condition *leisure* (a) and *work* (b)

Condition *leisure*	Condition *work*
You are buying a present in town and a tourist asks you for help. You also give tips on places of interest. The stranger is very grateful for your help and then you part.	You have a meeting with direct colleagues and talk about current projects. You also discuss problems and solutions to upcoming challenges in your everyday work.
Afterwards, you meet members of your family at a birthday party, gift the present you have brought and talk about everyday things. The atmosphere is relaxed.	You then have a meeting with unknown colleagues from another department and discuss project ideas and synergies. You immediately get along and then meet for a coffee.
You leave the party as you go on vacation next day and want to ask your neighbor a favor. While you are with your neighbor, you talk casually about your holiday destination.	Later, you have an important meeting with superiors. You discuss critical business decisions, the atmosphere is a bit tense. You present data about future developments.

#### Tasks & procedure

Our user study comprised two parts.

1. First, we asked participants to upload a frontal image of themselves with a neutral facial expression. We then created individual avatars for each participant and provided them with a unique access key.
2. Subsequently, participants engaged with their avatars throughout the study, which consisted of the following components:
  - an introduction to the system and experiment,
  - a demographic questionnaire,
  - the avatar selection, which included an explanation of the interactive features and a situational context (either *leisure* or *work*), and
  - a series of concluding questions.

In the demographic questionnaire, participants were asked about their age, sex, country, and job status. Subsequently, the participants were asked to experience specific topics: 3D computer games, creating 3D characters (e.g., *Sims* [72]), and VR. Additionally, participants were asked whether they would like to use new technologies. A short version of the Big Five Inventory (BFI-10) [73] was used to measure the participants’ personalities in five dimensions. Next, the participants were familiarized with the interactive features of avatar selection in a small tutorial. The interface consists of sliders to adjust the face geometry and texture material (Fig. 2).

After familiarizing themselves with the interface, the participants were presented with the situational condition and asked to adjust the avatar accordingly. The condition was presented with text and illustrations instead of videos, as the target group had limited time to participate in this study. Participants were then asked what their avatar would look like in such a situation. After selecting a specific avatar, two questions were asked regarding the situation and appearance of the avatar. The study was concluded with a set of questions.

**Fig. 4:**
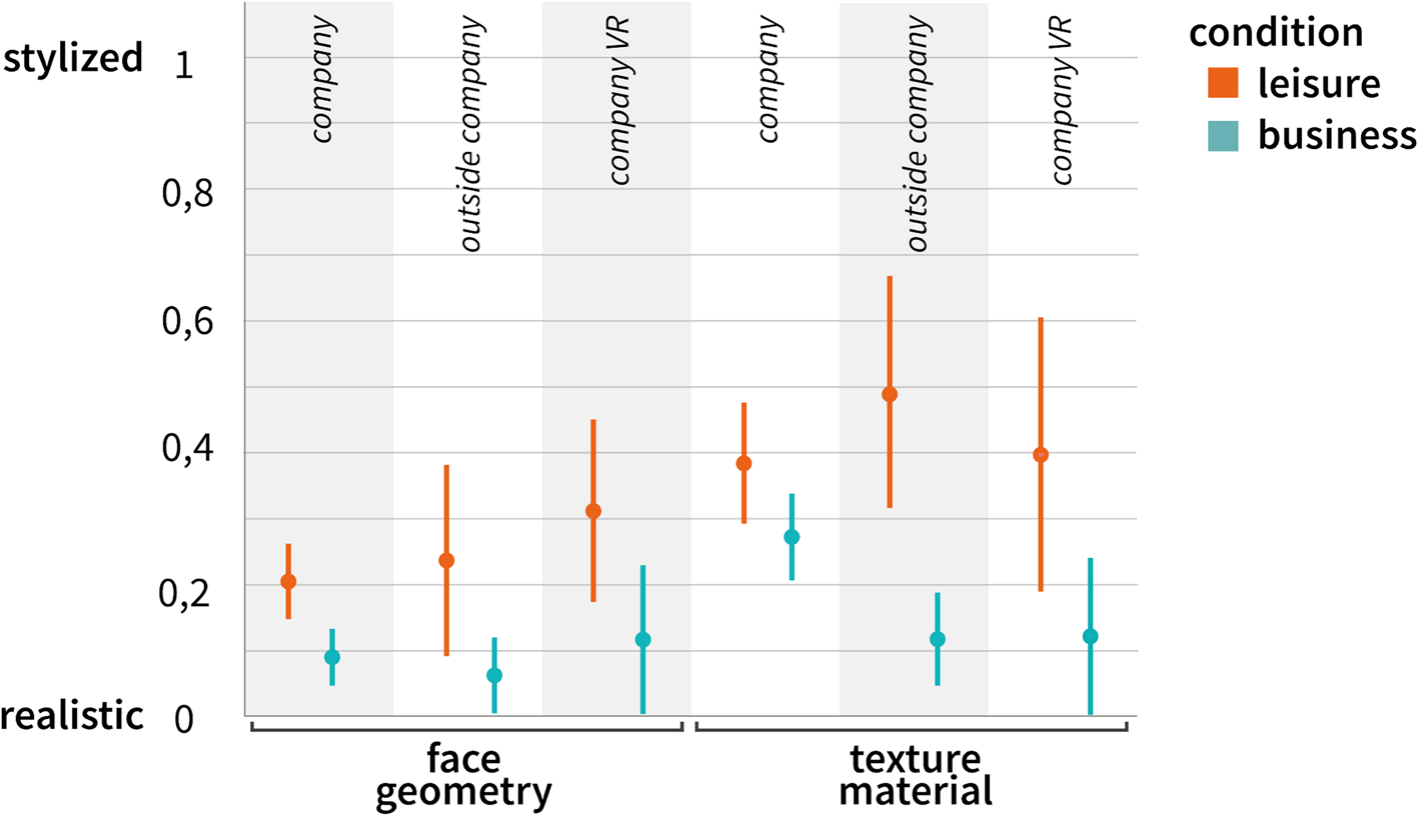
Face geometry (left) and texture material (right) based on the 95%CI for each condition for the degree of stylization *(0: lifelike - 5: stylized)*. The plots are divided into the different study setups: *company* (Study I), *outside company* (Study II), and *company VR* (Study III)

## User study I - company

In the primary study, the situational influence of selecting avatar representations was examined in *leisure* and *work* environments within the target group from an automotive company.

### Study design

The setup described in “Study design & setup” section was used.

#### Participants

The study included 71 participants (51 male and 20 female) from an automotive company. The goal was to investigate different avatar choices based on the presented situational condition (*leisure* or *work*) to understand the needs for avatar representations. The participants were recruited through mail lists and social networks. The mean age was 35.1 years, with range: 20-65 years). Ninety-six percent of participants stated that they frequently or very frequently worked on new technologies. On average, participants had a self-estimated experience of 3.89 ($SD = 0.96$; *1: very bad - 5: very good*) with VR and 3.62 experience using tools to create characters, for instance, in computer games 3.58 ($SD = 0.95$; *1: very bad - 5: very good*). The participants’ working areas ranged from engineering (requirement validation, data viewing, and ergonomics) to assembly planning and e-learning.

#### Design & variables

A between-subject study setup was designed. Conditions were randomly assigned, resulting in 35 participants being presented with the *leisure* condition and 36 with the *work* condition. The measured data included data collected from the slider interaction (face geometry and material texture), along with data from BFI-10 [73]. To gain further insight into the participants’ thought processes during the study, we incorporated a Likert scale questionnaire inquiring about perceived situational fidelity and avatar representation parameter adjustments. Subjective feedback were also collected from the participants through optional text fields.

#### Hypotheses

Based on the study design, the following hypotheses were proposed:

1. If users are in a *work* condition, the values for face geometry will be lower compared to the *leisure* condition.
2. If users are in a *work* condition, the values for the texture material will be lower compared to the *leisure* condition.
3. Personality, as measured by the BFI-10, will correlate with avatar choices in terms of texture material and face geometry.

### Results

The results including computed means and standard deviations are presented (Table 3, *company*). The effect sizes are depicted graphically with 95%CIs (Fig. 4, *company*).

#### Degree of stylization

The degree of stylization were measured under two conditions: *leisure* and *work*. We find evidence of an existing for the two conditions (Fig. 4). Because the directional hypotheses was formulated in *H1* and *H2*, the one-tailed *p*value of the two-sample t-test was used. In the *leisure *condition, the values show a higher mean value for *face geometry* than for the *work*condition (*95%CI* $[-0.97, -0.03]$, *t*(69) = 2.1, *p *= 0.019, *d *= -0.05). Similar results were obtained for the texture slider (95%CI $[-0.95, -0.007]$, *t*(69) = 2.02, *p *= 0.024, *d *= -0.48). The results show that in *work* situations, participants may choose a lifelike avatar in terms of face geometry and texture material compared to *leisure* situations, confirming *H1* and *H2*.

#### Measuring BFI-10

The participants were asked to answer a BFI-10 questionnaire to measure their personality and compare whether it was related to the choice of the avatar’s appearance. Looking at the different dimensions of the BFI-10, no personality trait showed a strong correlation with the preference for a specific *face geometry*.

**Fig. 5:**
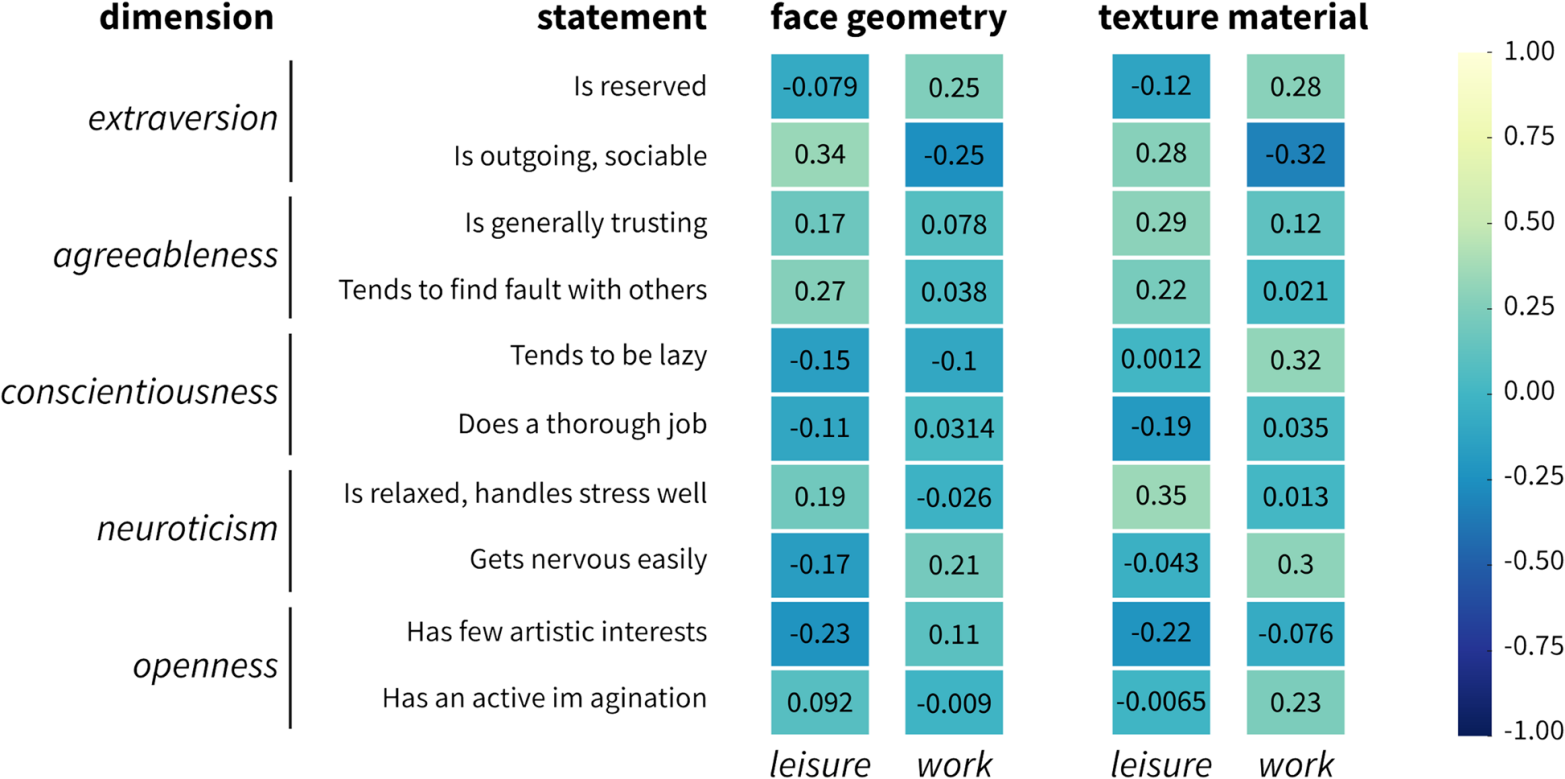
Correlation matrices showing the relationships between personality statements and avatar customization preferences between two conditions: *leisure* and *work*. The matrices illustrate correlation strengths, ranging from -1 (strong negative correlation) to 1 (strong positive correlation)

Additionally, a correlation analysis was performed to check whether correlations existed between individual personality statements. The analyses were conducted separately for the two conditions (Fig. 5). With regard to *face geometry* in the *leisure*condition, the personality statement *outgoing* showed a positive correlation, suggesting that more extroverted individuals may prefer more stylized avatar faces. This was the only statement in the *leisure*condition that showed a stronger positive correlation, directed towards a relationship between social extroversion and the preference for non-lifelike avatar representations. For the *work*condition, strong correlations were found. The lack of significant findings here may suggest that the factors influencing avatar selection in *leisure* situations differ from those in *work* situations, or are less related to the personality traits measured.

For *texture material*, it was found that in the *leisure* condition, the personality statement *relaxed under stress* showed a positive correlation with a preference for more stylized *texture material*, indicating that individuals who remain calm under pressure may opt for avatars with less lifelike textures in leisure environments. This preference suggests a desire to engage with the virtual world in a way that differs from reality, especially in contexts meant for relaxation and personal expression. However, no correlations were found in the *work *situation, indicating potential uniformity within professional settings. The need for professionalism and adherence to workplace norms may limit the expression of personal traits through avatar customization.

Although positive correlations between individual personality statements were found that form the basis for the dimension of personality traits, the results regarding personality traits, as measured by the BFI-10, suggest that they have limited influence on the preference for avatars and texture material. Thus, any statistically significant correlation between the degree of stylization and the personality dimensions assessed using the BFI was not found. Therefore, *H3* was not confirmed Nevertheless, it is believed that these results are noteworthy.

## User study II - outside company

To validate the results beyond the context of automotive companies, a follow-up study was conducted with 20 participants from the general population. To ensure comparability, the same study setup was used and only the differences in the following paragraphs were discussed.

In the main study, the differences in avatar appearance depending on the situation of the target group, an automotive company, were explored. In a follow-up user study, an exploratory methodology was adopted to investigate potential differences in avatar appearance choices regarding the examined parameters among participants not affiliated with an automotive company.

### Study design

Similar to Study I, a between-subjects design was used to compare the selected parameter values for both conditions. The conditions were equally distributed among the participants. In contrast to Study I, interest was in whether a specific company affiliation, in this case, that of an automotive company, influenced avatar choices based on the presented situation, and questions regarding the participants’ field of work were thus added.

#### Participants

Twenty participants (13 male and 7 female) were recruited using mail lists and social networks. The mean age was 31.9 years, with range: 20-59 years). Seventy percent stated that they work with immersive technologies frequently or very frequently. Participants had a self-estimated experience of 3.25 ($SD = 1.45$, *1: very bad-5: very good*) with VR and 3.55 experience using tools to create characters, for instance, in computer games ($SD = 1.4$,*1: very bad-5: very good*). Most (12 of 20) participants worked in the public sector or education system (e.g., as a teacher or university employee), and 8 of 20 worked in mechanical engineering or software companies. The other two participants stated that they worked in different fields.

#### Design & variables

The same conditions and measures as in Study I were used. It is hypothesized that, despite having a different field of work, participants do not choose a different avatar appearance compared to participants from within the company ($\text{H}_{\text{0}}$).

**Table 3:** Descriptive statistics for each group of participants

	Group	N	Mean	SD	SE
Face geometry	Company	71	0.1358	0.15090	0.01791
	Outside company	20	0.1455	0.17383	0.03887
Texture material	Company	71	0.3223	0.23592	0.02800
	Outside company	20	0.2970	0.26825	0.05998

**Table 4:** Bayesian factor for test with independent samples (method = Rouder). Unequal variance between groups is assumed. Bayesian factor: zero vs alternative hypothesis

	Mean difference	Pooled std. error difference	Bayes factor
Face geometry	0.0097	0.039510	5.094
Texture material	-0.0253	0.06156	4.851

**Table 5:** Posterior distribution characterization for the independent sample mean used in the Bayesian analysis in Study II

	Pasterior			95% credible interval	
	Mode	Mean	Variance	Lower bound	Upper bound
Face geometry	0.0097	0.0097	0.002	-0.0790	0.0985
Texture material	-0.0253	-0.0253	0.005	-0.1625	0.1120

### Results

Considering the small sample size, Bayesian Independent Sample Inference was used. The commonly used threshold for Bayes factors [74, 75], were used to interpret the results. The Rouder’s method was used to estimate the Bayes factor (Table 4). A Bayesian statistical analysis was performed for the avatar and texture sliders (Table 5). The Bayes factor for the face geometry parameters (5.094) and texture material (4.851) was between 3 and 10, indicating moderate evidence for a non-existent difference between the groups of participants. The effect sizes are graphically shown with 95%CIs (Fig. 4, *outside the company*).

## User study III - company VR

Because of the COVID-19 pandemic, conducting a study in person using VR headsets was avoided and a web-based study in which people could participate from home was conducted. Later, when it was safe to perform the in-person studies again, a follow-up study on VR with a subgroup of 17 participants was conducted from the main study. The goal of this study was to determine whether the display medium influences the choice of avatar. The study setup was kept the same and any differences are pointed out in the following sections.

### System description

This study is based on the avatar application introduced in Study I. The application was built using Unity and only minor adjustments were made by adding a neutral room to show the avatar and situation (Fig. 2b). The participants used an HTC Vive Pro 2 headset. To change the settings of the VR controllers, parameter sliders were adjusted using a laser pointer.

### Study design

In the present study, a shorter version of the questionnaire was used. Sections on demographics or personality were not included because that information had already been collected from the participants in the first study. Nine participants were presented with the *leisure* condition, and eight were presented with the *work* condition.

#### Participants

A group of 17 participants (11 male and six female) who had already participated in Study I were invited to participate in the follow-up study. The mean age was 31.8 years, with range: 20-59 years. The duration between Study I and this study was approximately two-four months. This part of the study was conducted as a supervised user study using HMDs, either in-person or remotely, via a conference system.

#### Design & measures

As every participant in the follow-up VR study had already participated in Study I, they were exposed to one condition. Consequently, in Study III, participants were assigned the opposite condition to prevent learning effects and to counterbalance the conditions. With this study setup, we aimed to identify the potential differences between immersive (VR) and non-immersive (desktop) environments ($\text{H}_{\text{0}}$).

### Results

Analogous to Study I, a two-sample t-test was used to compare both conditions of the VR users. Similar results were obtained: In the *leisure* condition, the values exhibited higher mean values for face geometry (*t*(15) = 2.5, *p*_2 _= 0.024) and texture (*t*(15) = 2.6, *p*_2 _= 0.021) compared to the *work* condition. The effect sizes are shown graphically with 95%CIs (Fig. 4, *Company VR*). When comparing the confidence intervals for both groups, we observed differing medians, which could be attributed to the smaller sample size compared with Study I. However, the tendency toward a more lifelike avatar for the *work* condition persisted for both parameter measures.

**Fig. 6:**
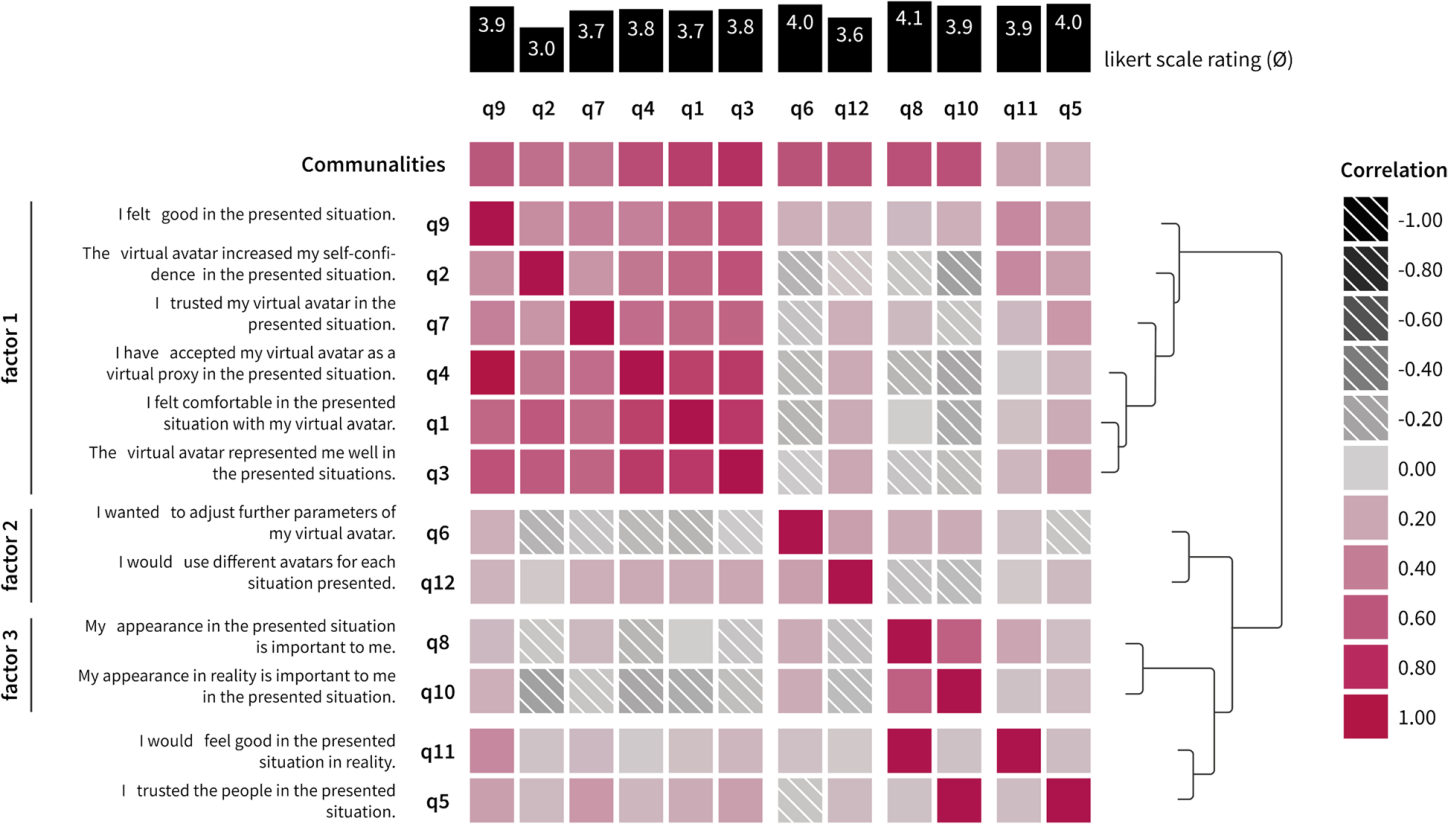
Correlation matrix of principal component analysis (PCA). Correlating items are visualized through a hierarchical dendrogram. Together with communalities in the top row and a scree plot (Fig. 7), three correlating factors that have an influence on the avatar selection were identified. Factor 1: Individual desires towards avatar appearance. Factor 2: Comfort in the presented situation through the avatar. Factor 3: Customization of the avatar appearance. *q11* and *q5* do not correlate. $N=91$

**Fig. 7:**
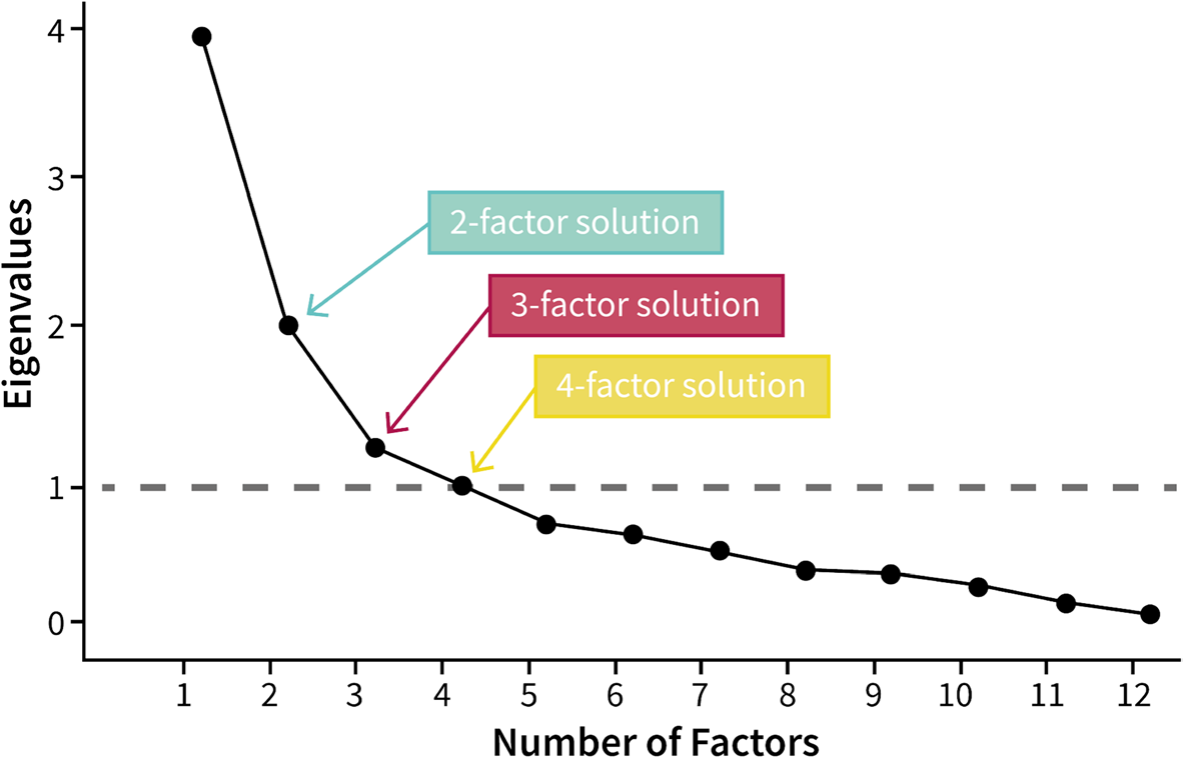
The scree plot shows the eigenvalue of factors and is used to determine the number of factors for the PCA. A thorough evaluation based on identifying the knee point and the interpretability of the data led to use of a three-factor solution

## Findings

To investigate the factors influencing the selection process for individual avatar appearances, a detailed examination was conducted of the collected data. Where appropriate, the resulting data were analyzed collectively.

### PCA

To uncover potential factors that could be interpreted as underlying motivational aspects of avatar appearance choices, PCA was used to analyze the questionnaire items (Fig. 6) using data collected in Studies I and II. PCA enables the analysis of multivariate data described by quantitative variables and visualizes information in tabular form ref. [76]. To ascertain whether the correlation of the items was strong enough, the Kaiser-Meyer-Olkin criterion and Bartlett’s test of sphericity were applied to assess the suitability of the data for performing PCA, and calculated measures of sampling adequacy [77, 78]. Based on the reviewed literature, the factors that were likely to be correlated were extracted. Consequently, the correlation matrix of all the items integrated into the PCA (Fig. 6) are presented. To determine the number of components to be retained for the PCA, a scree plot was used to visualize the eigenvalues of the correlation matrix (Fig. 7) and it was found that a two-, three-, or four-factor solution would be eligible. A subjective evaluation based on interpretability led to usage of a three-factor solution.

The oblimin rotation method ($delta=0$)was selected to extract the correlated factors, as empirically justified [79, 80]. Most questionnaire items loaded highly on one of the factors (Fig. 6, *communalities*). Only items *q5* ($h^2 = 0.23$) and *q11* ($h^2 = 0.17$) exhibit no correlation with the other variables. Variables should be intercorrelated and must have at least one correlation equal to or greater than 0.3. The three-factor solution displays an interpretable matrix of loadings (Fig. 6) to describe the potential factors that influence avatar appearance selection. It is proposed that these factors form the foundation for adapting and accepting one’s avatar in a VE: (1) *individual desires towards avatar appearance* that increase acceptance, (2) *comfort in the presented situation through the avatar*, and (3) *customization of the avatar’s appearance*.

### Gender differences in avatar selection

**Fig. 8:**
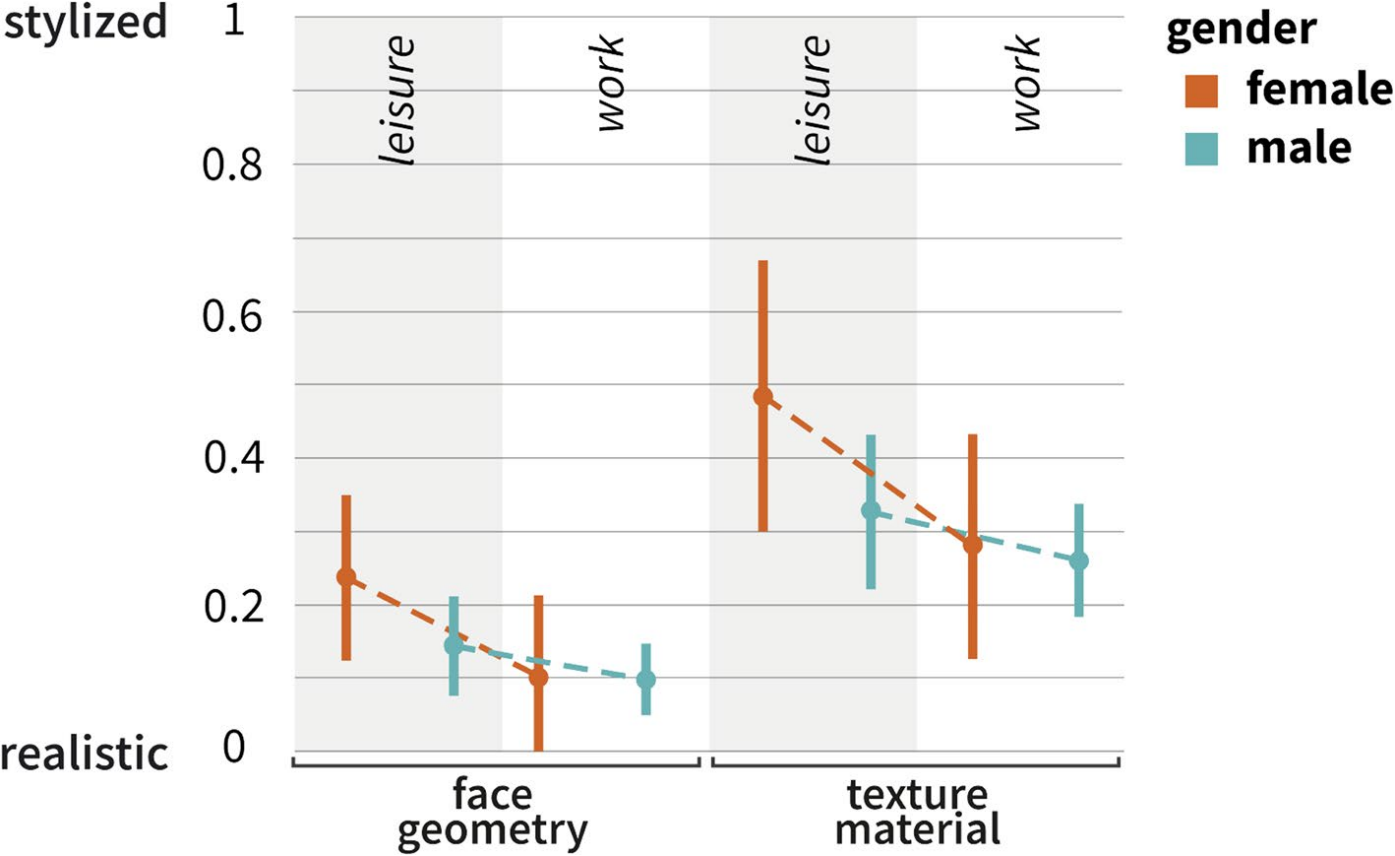
Face geometry (left) and texture material (right) based on the 95%CI for internal male and female participants for both conditions (Studies I and II). $N=91$

To investigate potential differences between male and female study participants, a two-sample t-test that included both groups of participants was performed. For face geometry, the t-test revealed a difference in mean preferences between genders in the *leisure* condition, with *p*_2 _= 0.057), indicating a trend towards significance. The mean face geometry values for males and females under this condition suggested a slight preference for more stylized avatars among females (Fig. 8). By contrast, under the same conditions, the texture material resulted in *p*_2 _= 0.152), indicating no significant gender-based differences in preferences for the material used in the *leisure* condition. For the *work* condition, the two-sample t-test resulted in *p*_2 _= 0.891) for face geometry and *p*_2 _= 0.993) for texture material. These results show a lack of significant gender differences in preferences for both avatars and texture stylization in the work context.

**Fig. 9:**
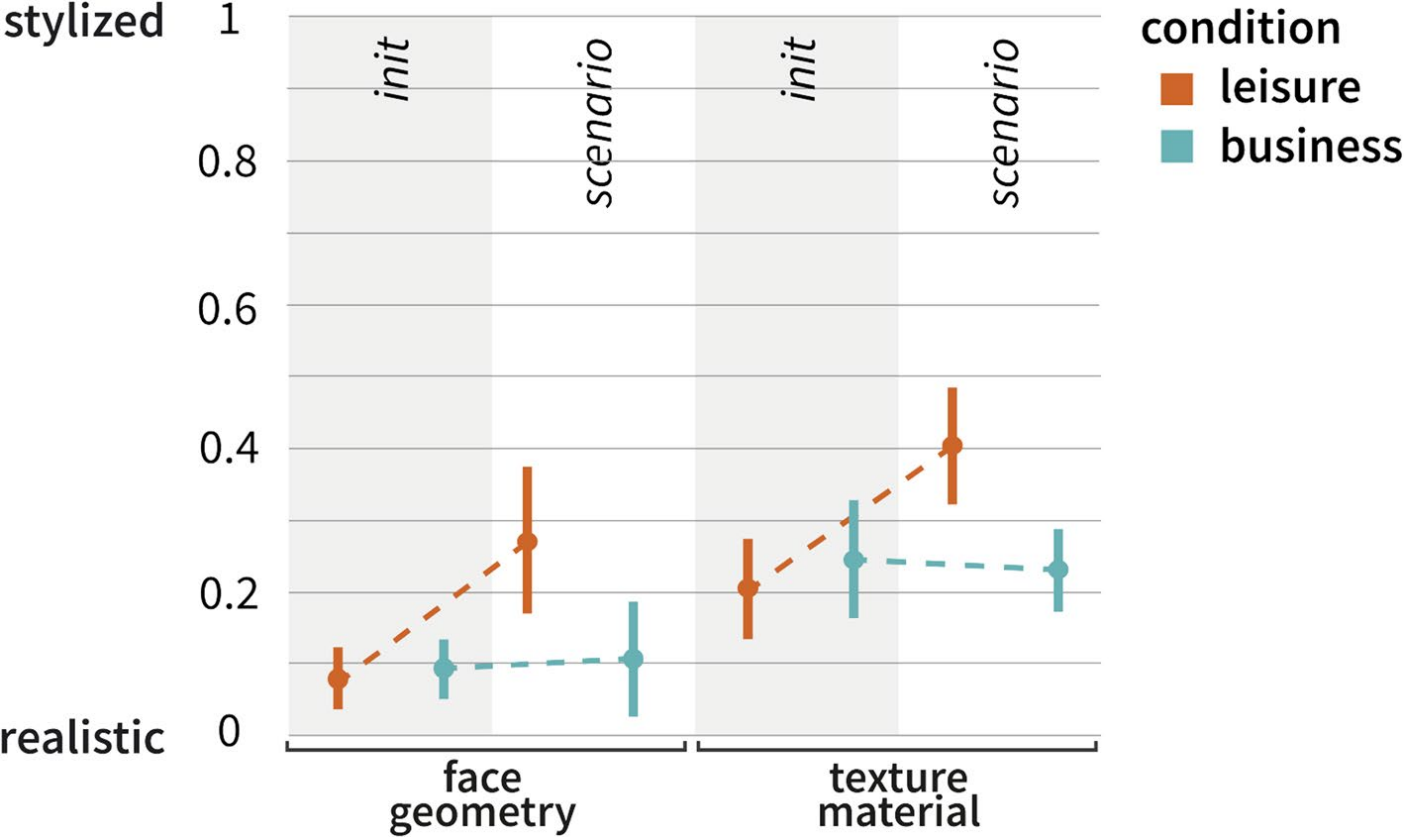
Plots based on 95%CIs visualize a potential interaction effect between the initial avatar selection and the selection for the given situation based on condition *leisure* or *work*. The plots are computed from the data of all participants ($N = 91$)

### Interaction effects

Each participant in Studies I and II was presented with a tutorial explaining the tool’s interactive features. At the conclusion of the tutorial, participants were asked to adjust the avatar in a manner that they deemed *suitable*. The results of the participants’ initial inputs were compared during the tutorial with their inputs for the scenario. Visual exploration of the graphs implies that a potential interaction effect may exist (Fig. 9) when deciding on an avatar representation. The plot reveals that stylization levels are marginally higher for the *leisure* condition. It is surmised that this is because of the more playful nature of the situations in the *leisure* condition.

**Fig. 10:**
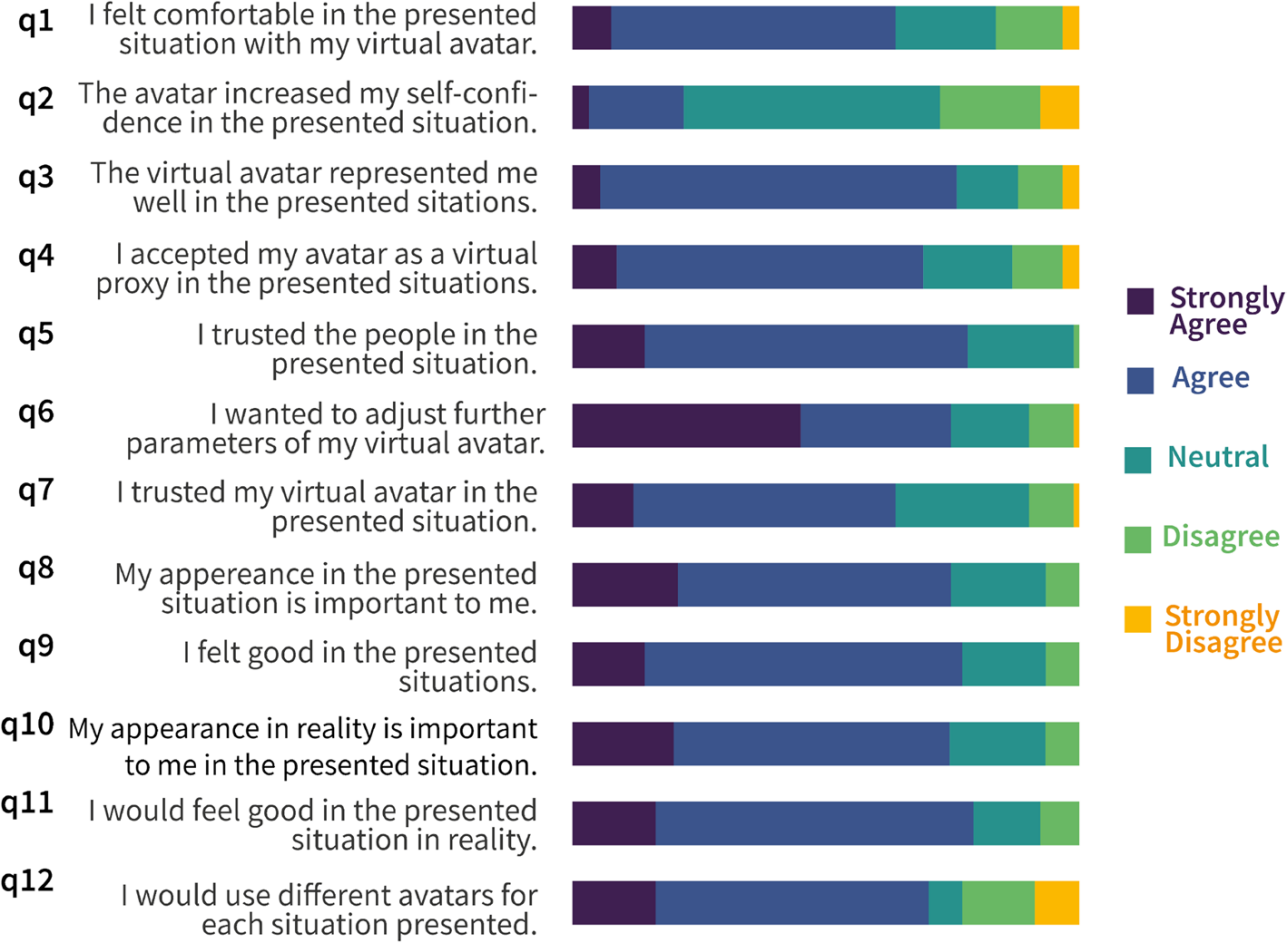
Results of Likert questionnaire after participants selected their avatar for the presented condition. $N=91$

### Subjective feedback of the avatar in the presented situation

Interest was also in the extent to which participants identified with their individual avatars and their satisfaction with the representation of the presented situation. As evidence of a difference between participants from Studies I and II, were not found, the results of the Likert scale responses and report them in Fig. 10. The Likert scales indicated that participants rated their satisfaction with and acceptance of their virtual avatar in the presented situation. By comparison, statements regarding the increasing effect on self-confidence (item *q2*) received lower ratings. It is speculated that the lower ratings are due to the fact that overall, participants already experienced an increased feeling of comfort in the presented situation, and thus, the avatar’s appearance *“neither positively nor negatively influenced”* their self-confidence.

### Qualitative feedback

A simplified version of thematic analysis was used [81]. This process involves reviewing comments, creating initial codes, and identifying themes within the codes.

**Fig. 11:**
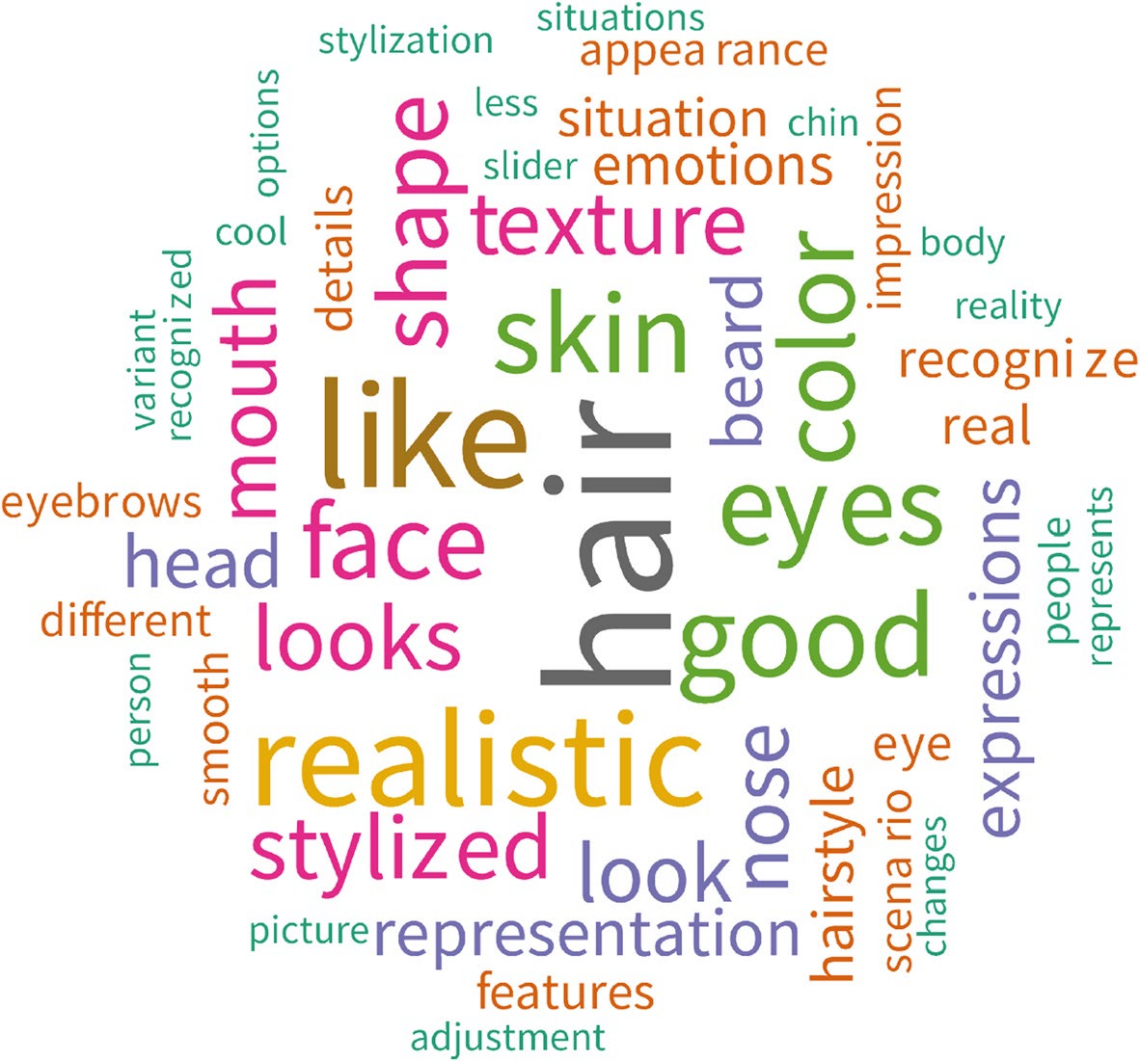
Word cloud visualizing the users’ feedback, highlighting the main topics mentioned in their responses

From the subjective feedback collected in the form of optional free-text fields from each participant, it was found that customization was frequently mentioned in the 580 comments collected from all participants. In addition, a word cloud was computed to visualize the most frequently used words in the comment collection (Fig. 11). Here, note that the avatar is based on a single frontal image; thus, the resulting avatar does not have a full-hair mesh. This information was communicated to the participants in advance. The analysis of the feedback showed that participants had specific likes and dislikes of avatar design, expressing a need for personalization and lifelike portrayals in virtual settings. The comments mainly focused on the desire to adjust the facial features and improve the representation of their avatar’s facial features.

Almost 75% of the participants expressed a desire to adjust additional avatar parameters. All the comments targeting the adjustment of additional facial features are summarized and grouped into corresponding topics. Most comments (21%) expressed a desire to adjust their (facial) hairstyles and hair color. These comments were followed by requests to modify the face shape (12.6%), eye shape (11.9%), skin texture (8.3%), mouth features (7.4%), nose (4.5%), and ears (2.1%). Additionally, 4.1% of the comments expressed a desire for further modifications, such as makeup, jewelry, and glasses.

These results highlight the importance of offering diverse avatar customization options to cater to a broad spectrum of user preferences, thus enhancing individuals’ experiences and their interactions within virtual settings.

## Discussion

The investigation provides valuable insight into the choice of avatar representations in CVEs. The findings from the three studies in Table 1, are summarized and an extended discussion of how these outcomes intersect with the existing literature is delved into, and implications for design and practice offered.

### User preferences for avatar selection shifts with context

The study suggests that the context in which the avatar is used may influence the selection of facial geometry and texture material, affirming previous studies that suggested user behavior modulation based on the situation [82]. The results of the studies were as expected; however, they provided empirical evidence that, in different situations, users tend to choose a different avatar fidelity. This finding suggests that human-like feature avatars are preferred in professional settings, as users may not wish to appear as cartoon characters in front of colleagues or superiors [83]. This context dependency aligns with the Social Identity Model of Deindividuation Effects theory [84], emphasizing the social influence of contexts on self-representation. While research has largely focused on abstract vs anthropomorphic avatar designs, the study provides evidence to guide life-like avatar design in work settings. To align CVE design with user preferences, designers should consider context-specific avatar customization options.

The importance of avatar realism in enhancing the sense of embodiment and presence suggests that engaging and immersive VEs are crucial for effective communication and collaboration, particularly in professional contexts [85]. Furthermore, the ability to customize avatars allows users to express their identities, thereby affecting their social interactions and overall satisfaction within these virtual spaces [86].

Considering the impact of avatar design on the perceptions of professionalism [87] and recognizing the importance of cultural differences in avatar preferences [88], future CVE designs must offer context-specific avatar customization options. This approach not only supports the integration of professionalism and personal expression, but also addresses cultural inclusivity in global virtual teams.

The exploration of adaptive avatars that adjust their appearances based on contextual cues is a promising avenue of research. Such adaptiveness can enhance the user experience by ensuring situational appropriateness and personalization, a concept supported by emerging technologies such as artificial intelligence and machine learning [89].

It is believed that matching avatar designs to the context in which they are used-whether for work or leisure-is important for CVEs. The discussion highlights the need for future research to examine how different contexts influence avatar preferences. This can help improve virtual worlds for users, enhancing their overall experience. It is also believed that it is important to match avatars to their use, such as for work or play. Thus, it is suggested that research be conducted on how setting affects the choice of an avatars people choose. This can help improve virtual worlds for everyone.

### Influence of display medium

The exploratory results of Study III added a new dimension to the discussion: the possible effect of display media on avatar selection. This insight agrees with studies indicating distinct user behaviors between desktop and VR environments [90–92]. VR’s immersive nature potentially amplifies the context effects on avatar selection, a consideration that CVE designers should consider when building systems intended for different display media.

Research comparing immersive and non-immersive environments suggests that immersive VR qualities affect user preferences and behaviors, including avatar selection [93]. This is further supported by studies on presence and embodiment, where a heightened sense of being in the environment and identification with an avatar in VR may lead users to prefer avatars that augment their feelings [94]. Additionally, the differences in user experience and performance across display media underscore VR’s unique capacity of VR to offer engaging experiences, potentially influencing users to select avatars that enhance immersion [95].

Given the preliminary nature of the findings and the small sample sizes in Studies II and III, these insights should be interpreted with caution. However, they pave the way for future research to explicitly examine how different display media, from traditional desktop interfaces to immersive VR and augmented reality (AR) technologies, affect avatar selection behavior. Such studies are essential for developing CVEs that are sensitive to the nuances of display-medium influences, aiming to create more engaging and responsive VEs for users.

### Factors determining avatar appearance

The analysis illuminates three major factors that influence avatar appearance choice: (1) individual desires, (2) comfort in the situation, and (3) customization. This aligns with Self-Perception Theory [96], which suggests that people develop their attitudes (including self-representation) by observing their own behavior and considering what must have caused it.

#### Individual desires

Individual desires reflect the motivation to enhance self-presentation, which is consistent with studies discussing users’ tendencies to adjust to unflattering avatar features. For example, the appearance of an avatar can influence user behavior in virtual interactions [97], potentially leading users to prefer avatars that enhance their self-esteem or project an ideal self-image. This highlights the need of providing many options to customize their avatar, so they can either make them look like themselves or improve their appearance [82, 98, 99], as the desire to adjust features was mentioned multiple times, as one participant phrased, *“The nose was a bit big, but maybe I just have a big nose.”*, and another one stated *“My wrinkles tend to look worse [due to age].”*

Developers should focus on creating advanced and easy-to-use customization tools, allowing users to adjust their avatars in detailed ways. It is believed that this approach not only enhances user satisfaction but also helps foster a stronger personal connection and sense of identity in the CVE.

#### Comfort in the situation

In this situation, comfort is the key to choosing avatars. Research shows that people consider how comfortable they feel in a virtual world before customizing their avatars [100]. This feeling of comfort affects their choices, highlighting the need for avatars that make users feel relaxed in VE. Studies have also found that the appearance of an avatar can change how people act, such as in virtual gambling games [101]. Research has also discovered that the appearance of avatar pretouch proxemics in VR affects users’ spatial comfort levels [102], and thus, how comfortable people feel about their personal space in VR.

Designers should focus on creating avatars that help users feel safe and like they belong to these VEs. Future studies should examine the psychological impact of avatar appearance on user comfort and explore how different designs affect users’ willingness to engage in and interact with CVEs.

#### Customization

Customization addresses the desire for dynamically adjustable features, such as hair and accessories, to make their appearance more personal. This is important for creating avatars as it allows users to create avatars that truly represent a user’s identity and helps increase the feeling of presence in CVEs. Offering advanced tools for users to adjust and detail their avatars to match how they see themselves enhances their connection with and satisfaction with their avatars [82, 98, 99].

Being able to change avatars on the go is important too, as it lets users reflect any new preferences or life changes, such as accessories or hair, as these can be rather volatile in reality: *“I would like to adapt my hair style and color, as it often changes.”* Four participants stated that adjustable features can help increase their perceived closeness towards their own avatar representation to make it feel as if it was indeed their own. This flexibility makes users feel more connected to their avatars and immersed in the virtual world [103].

Research on how customization affects user engagement and comfort will help us understand how different features affect user actions and socialization in virtual spaces, thereby guiding the creation of better virtual experiences [86, 88, 104].

Developing detailed guidelines for avatar customization will help to meet the varied needs of users, leading to more engaging and satisfying experiences in different virtual settings. Thus, customization is a fundamental element of avatar design and is important for enhancing the user experience in VEs.

### Personality and avatar choice

The findings revealed no significant correlation between the Big Five personality traits and avatar selection, which is consistent with some studies [105, 106] but conflicting with others [38, 107–109]. This discrepancy underscores the complexity of the impact of personality on avatar choices and calls for further investigation.

The choice of an avatar and personal identity in a VE is an area of study that examines how users project their identities through avatars [86, 110]. In leisure activities, people who are outgoing or relaxed under stress tend to choose avatars that look less lifelike, showing a preference for creativity and as a way to escape reality. Research on how extroverted individuals engage with online platforms suggests that they may choose more expressive and stylized avatars to facilitate social interactions [111, 112]. This preference could explain the positive correlation between extroversion and stylized avatar preference in leisure settings, demonstrating the potential role of personality in adjusting avatars for VEs.

However, the overall lack of strong correlations in the work condition suggests the potential constraints that professional norms place on avatar customization, potentially moderating the expression of personality traits through virtual representation. This observation aligns with the broader discourse on online identity and self-presentation, indicating the distinction between how personal and professional identities are managed in VEs.

Based on these insights, it is recommended that leisure platforms offer more options for creative avatar customization to improve user enjoyment. For work-related VEs, designs should allow for some personal expression but remain within professional standards, perhaps through customizable templates that maintain a professional look.

### Potential gender-based preferences in avatar selection

Although a statistically significant overall gender difference, was not found, it is worth noting that a potential trend exists towards a gender-based difference for the preferred texture material in a leisure context. This trend aligns with previous research, suggesting that avatar choices may reflect deeper aspects of identity expression and social interaction in VEs [113, 114]. In this context, researchers have discussed the importance of anonymity, suggesting that stylized avatars can serve as a means of navigating online spaces more freely, particularly for female users seeking to avoid gender stereotyping [88]. In addition, online games and social platforms can be tools for exploring one’s identity, as users often experiment with appearances that differ from their real-world selves [115]. A study exploring gender differences in creating attractive and unattractive avatars found that females may lean towards more stylized avatars than males, potentially as a strategy for navigating online spaces with greater freedom and avoiding gender stereotyping [116]. Moreover, avatars can facilitate identity exploration and social categorization, including gender [29]. This supports the notion that stylized avatars can offer users, particularly females, opportunities to experiment with their identities in ways that diverge from their real-world selves, which is aligned with the observations in leisure contexts.

This potential trend towards gender-based preferences in avatar selection suggests underlying factors that warrant further exploration. This highlights the importance of sex in designing avatars and VEs to accommodate diverse user needs and preferences.

## Limitations

As with all user studies, the applicability of the results is limited to the specific stimuli and settings employed in the research. Focus was on the distinct styles of lifelike and stylized avatars, which may have constrained the findings to these particular types. Nonetheless, because of the thorough research and design of the avatars, the conclusions regarding the level of stylization can be believed to be relevant to avatars with similar facial geometries and textures. These preferences were observed across diverse display media and work backgrounds under controlled experimental conditions involving 91 participants from various working backgrounds, mainly automotive. Given the sample size and study setup, the generalization of VR avatars was not fully supported by Study III. The results indicated no significant differences. regarding the used situational conditions in Studies I and III, a minor tendency towards an increasing effect of VR between both conditions exist (Fig. 4). The facial expressions are included on demand, while adjusting for sliders of their avatars to show how their avatars’ emotions are displayed. Exploring the animations Applied to external avatars was outside the cope of this study Here, it is expected that there may be an interdependence between motion and avatar representations, and researchers may want to look into this in future studies. However, the study setup did not allow for generalization over all situations and people in different fields of work. Instead, lifelike avatars in terms of the investigated parameters and situations are advocated.

## Implications & future directions

While research has largely focused on abstract vs anthropomorphic avatar designs, this study provides insights to guide the design of avatars that users might prefer in professional settings based on their context-specific functionalities. The findings contribute to the understanding of user preferences in avatar representation and the design of avatar-supported CVEs. We advocate context-specific design approaches reinforced by extended customization options that factor in the individual desires and comfort of the users. In addition, it is believed that personalized avatars could make people more open to using VEs for work. In the future, examination of the use of multiple avatars in work settings to learn more about how people choose to represent themselves should be carried out. Here, interest is also in this feeling of co-presence, as this is key to forming social connections. Another point to explore is to give users more control over customizing their avatars. The avatar-making tool should be updated to include more options for users to tweak their avatars’ appearance. Several directions are proposed for future research in this field.

Exploring influences on avatar selection, such as cultural background and various work situations should be explored. Future studies could examine how avatars can help people accept and use VEs in work-related scenarios. This includes studying self-representation, co-presence, and their impact on social relationships despite the lack of significant differences between internal and external company participants.

Studying how the quality of avatars affects teamwork in virtual workspaces can help to understand what makes up a good workplace avatar. This knowledge can lead to improved avatar design rules for virtual workspaces, thereby making online work more enjoyable and productive.

Emerging technologies like artificial intelligence and machine learning should be explored to make avatars more adaptable. This can lead to avatars that automatically change based on the situation, thereby enhancing the virtual workspace experience.

Although demographic influences and personality traits did not show a strong correlation between avatar selection and participant age or occupation, future research could specifically target these demographic factors. In addition, investigating the influence of personality traits on avatar customization choices could offer deeper insights into user preferences.

Considering gender differences in avatar selection can provide valuable insights into behaviors in virtual spaces. Future studies with more or different types of participants could help to better understand these differences and help to make avatar designs more inclusive for everyone.

## Conclusions

In this study, the situational impact on choice of the avatar representation was examined and three user studies were conducted (Table 1) with 91 participants. Specifically, two different situational contexts, work and leisure contexts, and individual virtual avatars with which the participants participated in the study. The data were statistically analyzed and qualitative feedback collected. With regard to the selection of the avatar appearance, three correlations Factors were revealed: (1) individual desires, (2) comfort in the situation, and (3) customization. Any evidence of users’ personality traits influencing avatar selection was not found. Overall, the results show that, in contrast to most consumer applications, participants choose more lifelike avatars in terms of face geometry, and textural materials when situated in a work context. in the leisure context regardless of the display medium. It is believed that CVEs that implement lifelike avatars can improve the overall immersive Experience, especially in a world where work meetings are shifting to take place remotely, with an increasing number of home offices.

## Data Availability

The datasets used and/or analyzed in the current study are available from the corresponding author upon reasonable request.

## Abbreviations

CVE: Collaborative virtual environment
VE: Virtual environment
VR: Virtual reality
BFI: Big Five Inventory
HMD: Head mounted display
PCA: Principal component analysis
